# Lysosomal cathepsin creates chimeric epitopes for diabetogenic CD4 T cells via transpeptidation

**DOI:** 10.1084/jem.20192135

**Published:** 2020-10-23

**Authors:** Brendan Reed, Frances Crawford, Ryan C. Hill, Niyun Jin, Janice White, S. Harsha Krovi, Philippa Marrack, Kirk Hansen, John W. Kappler

**Affiliations:** 1 Department of Biomedical Research, National Jewish Health, Denver, CO; 2 Department of Immunology and Microbiology, Anschutz Medical Campus, University of Colorado, Aurora, CO; 3 Research Division, Barbara Davis Center for Diabetes, Anschutz Medical Campus, University of Colorado, Aurora, CO; 4 Biochemistry and Molecular Genetics, Anschutz Medical Campus, University of Colorado, Aurora, CO

## Abstract

The identification of the peptide epitopes presented by major histocompatibility complex class II (MHCII) molecules that drive the CD4 T cell component of autoimmune diseases has presented a formidable challenge over several decades. In type 1 diabetes (T1D), recent insight into this problem has come from the realization that several of the important epitopes are not directly processed from a protein source, but rather pieced together by fusion of different peptide fragments of secretory granule proteins to create new chimeric epitopes. We have proposed that this fusion is performed by a reverse proteolysis reaction called transpeptidation, occurring during the catabolic turnover of pancreatic proteins when secretory granules fuse with lysosomes (crinophagy). Here, we demonstrate several highly antigenic chimeric epitopes for diabetogenic CD4 T cells that are produced by digestion of the appropriate inactive fragments of the granule proteins with the lysosomal protease cathepsin L (Cat-L). This pathway has implications for how self-tolerance can be broken peripherally in T1D and other autoimmune diseases.

## Teaser

The lysosomal protease cathepsin L uses pancreatic secretory granule protein precursors and a reverse proteolysis mechanism, transpeptidation, to generate chimeric super-epitopes specific for diabetogenic CD4 T cells.

## Graphical Abstract

**Figure:**
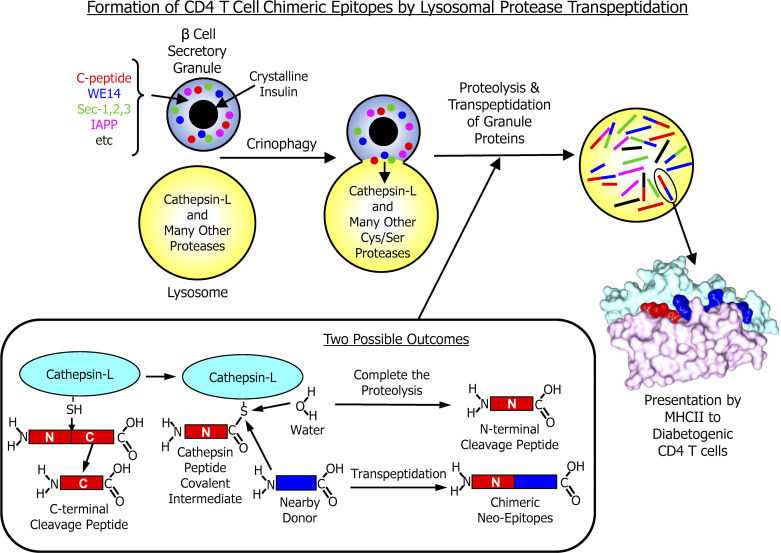

## Introduction

Identification of the CD4 T cell peptide epitopes driving autoimmune diseases has been surprisingly difficult over the years. The original discovery that particular MHC class II (MHCII) alleles were strongly associated with the development of many of these diseases in rodents and humans (reviewed in Nepom and Erlich, 1991) led to the idea that peptide epitopes derived from the target tissue–specific proteins presented by these MHCII alleles might be the CD4 T cell targets in the diseases. However, even when a stimulatory protein or peptide was identified, responses to it were often very weak. Eventually, in some cases, much stronger epitopes were identified that had enzymatic posttranslational modifications of particular amino acids in the natural peptides (Sollid and Jabri, 2011; Wegner et al., 2010).

Type 1 diabetes (T1D) is an autoimmune disease caused by immune destruction of the insulin-producing β cells within the pancreatic islets of Langerhans, leading to a lifelong dependence on daily administrations of insulin. In rodents and humans, certain MHCII alleles (IA^g7^ in mice, RT2^u^ in rats, and HLA-DQ8 or HLA-DQ2 in humans) pose a high risk for developing the disease (reviewed in Marrack and Kappler, 2012; Mullen, 2017). Over several decades, numerous CD4 T cell clones in mice and humans have been identified whose MHCII-presented pancreatic epitopes map to several pancreatic β cell granule proteins; however, as with other autoimmune diseases, natural peptides derived from these proteins were often either inactive or only weakly active in stimulating the clones (Dallas-Pedretti et al., 1995; Stadinski et al., 2010a,b; Wang et al., 2018). Recently, we and others have reported highly stimulatory versions of these peptides in which certain amino acids in the natural peptide have been replaced with other amino acids sometimes derived from the same or different pancreatic proteins (Babon et al., 2016; Crawford et al., 2011; Delong et al., 2016; Jin et al., 2015; Stadinski et al., 2010a,b; Wang et al., 2018, 2019; Wiles et al., 2017; Yang et al., 2014). In a few cases, these chimeric peptides have been shown to be present in the pancreas itself (Wiles et al., 2017, 2019).

An in vivo mechanism for generating these chimeric peptides for MHCII presentation has not yet been identified, but recently the generation of chimeric epitopes for MHCI presentation to CD8 T cells has been shown to occur in the proteasome via a version of reverse proteolysis called transpeptidation, mediated by the proteasomal threonine proteases (Faridi et al., 2018; Hanada et al., 2004; Liepe et al., 2016; Vigneron et al., 2004). Transpeptidation, a phenomenon initially identified >80 yr ago, is common in nature and can occur with proteases that form a transient covalent bond to the new carboxylate at the cleavage site (reviewed in Berkers et al., 2009; Cresswell, 2004). This bond can be broken by water to complete the cleavage or by the N terminus of another nearby peptide to reform a peptide bond and a new chimeric peptide or protein.

We have argued (Jin et al., 2015; Wang et al., 2018, 2019) that transpeptidation in the lysosome/endosome compartment is a likely mechanism for generating chimeric epitopes for MHCII, since these organelles contain many cysteine/serine proteases capable of mediating the reaction and, moreover, are the location where most peptides destined for presentation by MHCII are produced. Furthermore, in β cells and other neuroendocrine cells, secretory granules that contain high concentrations of the relevant protein precursors of potential autoantigen epitopes continuously turn over by the process of crinophagy, in which the granules fuse with lysosomes and their contents are degraded by these proteases (Boudier and Picard, 1976; Sandberg and Borg, 2006).

To test these ideas, we exposed fragments of β cell granule proteins that, in their natural form, stimulated diabetogenic CD4 T cells weakly or not at all, to several lysosomal cysteine proteases under lysosomal conditions. We found that the cysteine protease cathepsin L (Cat-L) mediated a transpeptidation reaction in vitro between incomplete epitopes derived from chromogranin A (ChgA) or islet amyloid polypeptide (IAPP) as donors with acceptors derived from the same or other pancreatic proteins, thereby creating highly stimulatory chimeric peptides for diabetogenic mouse CD4 T cells. Tandem mass spectrometric (MS/MS) analyses of these digestion reaction products revealed the presence of not only the predicted stimulatory chimeric epitopes, but also scores of other chimeric peptides. These data suggest that the lysosome, like the proteasome, can be an efficient site for posttranslational modification by transpeptidation, leading to the creation of neo-epitopes for MHCII presentation. Since transpeptidation requires a high concentration of the donor peptide to compete with water, our findings presented here may explain how autoreactive diabetogenic CD4 T cells escape negative selection in the thymus, where the precursor proteins are at low concentrations, but subsequently encounter their optimal stimulatory epitope in the pancreas.

## Results

### Identification of amino acids fused to the N terminus of WE14 that create super-agonists

**Figure 1.**
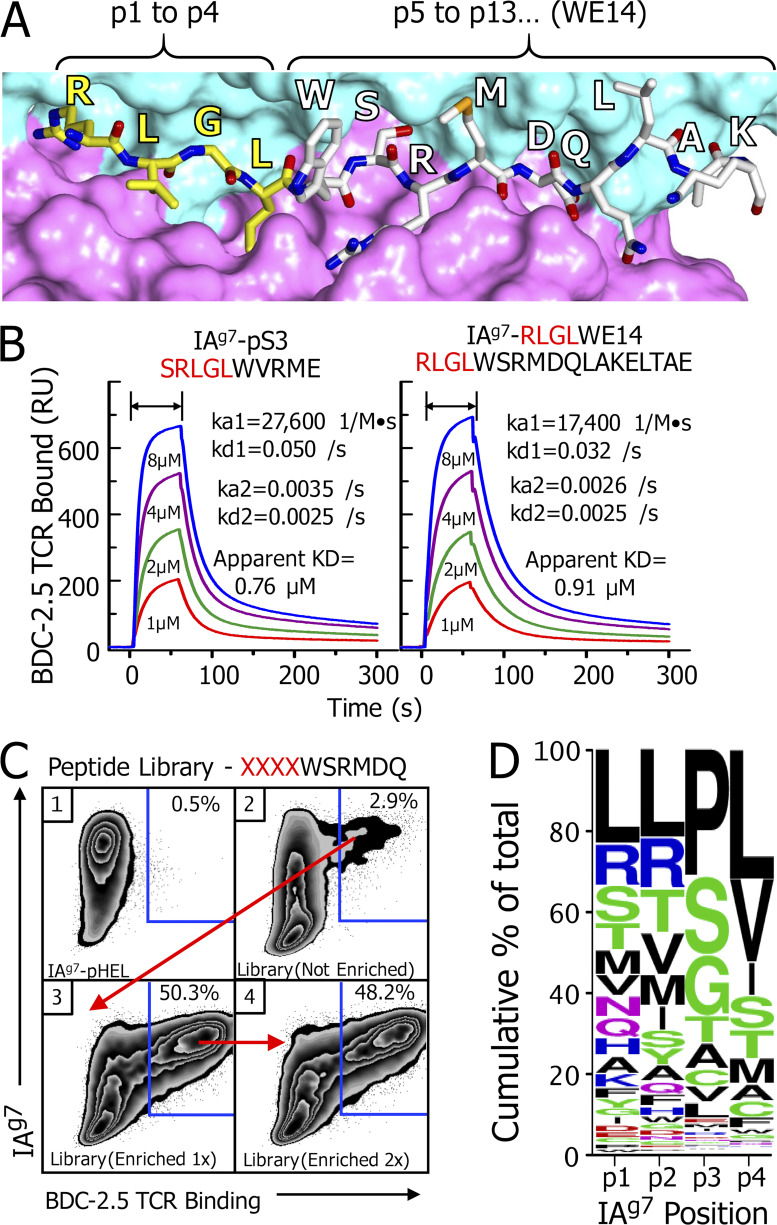
**Sequences added to the Nterminus of WE14 that create super-agonists for BDC-2.5 and other WE14-specific T cells. (A)** View of the crystallographic structure of the RLGLWSRMDQLAKELTAE (RLGL–WE14) chimeric peptide bound in the groove of IA^g7^ (Jin et al., 2015). The surface of the IA^g7^ α (cyan) and β (magenta) chain helices flanking the peptide binding groove are shown. A wireframe representation of the peptide in the groove is shown with the amino acids labeled. Carbons in the RLGL portion of the peptide are yellow, and in the WE14 part, white. Brackets indicate the relative positions of the two parts of the peptide in the groove. **(B)** SPR signal (in RU) obtained with various concentrations soluble BDC-2.5 TCR during binding to (arrows) and dissociation from immobilized IA^g7^ covalently attached to either the SRLGLWVRME (left) or RLGL–WE14 (right). Data were corrected for the fluid-phase SPR signal using IA^g7^ bound to an HEL peptide. Kinetic constants were calculated for a two-state (conformational change) model with BIAcore BIAevaluation 4.1 software. **(C)** SF9 insect cells were analyzed for binding both an anti-IA^g7^ Mab (OX-6) and a soluble multimer of the BDC-2.5 TCR. The SF9 cells were infected with baculovirus expressing control IA^g7^-HEL peptide complex (1) and baculovirus expressing IA^g7^ bearing an unenriched library of peptides consisting of a randomized tetrapeptide (XXXX) followed by the WE14 fragment, WSRMDQ (2). The infection and analysis were repeated with baculovirus prepared from the sorted double binding cells in panel 2 (blue gate; 3). The infection and analysis were repeated with baculovirus prepared from the sorted double binding cells in panel 3 (blue gate; 4). **(D)** Baculoviral DNA from the viral stock prepared from sorted cells in C, panel 3, was used as a template to create a PCR fragment containing the sequences of the enriched peptides. The fragment was sequenced, and the results were used to create a Weblogo (Crooks et al., 2004) summary of the highest-frequency amino acid usages at the p1 to p4 positions as enriched by the BDC-2.5 TCR. See text and Data S1 for more details.

WE14 is a natural 14–amino acid cleavage product of ChgA. We and our collaborators identified WE14 as a weak agonist for the prototypical BDC-2.5 and other ChgA-specific CD4 T cells some years ago (Stadinski et al., 2010a). However, comparison of the WE14 sequence to that of strong agonists for these T cells identified by us and others in peptide libraries (Judkowski et al., 2001; Stadinski et al., 2010a; Yoshida et al., 2002) strongly suggested to us that the WE14 peptide was an incomplete epitope binding to IA^g7^ with only its N-terminal five amino acids occupying the p5 to p9 positions in the IA^g7^ binding groove, leaving positions p1 to p4 empty. Adding back the four natural ChgA amino acids (EDKR) upstream of WE14 did not improve the peptide activity but rather destroyed its weak stimulatory activity altogether (Stadinski et al., 2010a), so we concluded that some other addition was required. To test this idea (Crawford et al., 2011; Jin et al., 2015), we replaced these four natural ChgA amino acids with four amino acids, RLGL, from our library super-agonist, pS3 (SRLGLWVRME; Stadinski et al., 2010a). This peptide was >100,000× more potent than WE14 in stimulating a variety of ChgA-specific T cells. Also, fluorescent IA^g7^ tetramers bearing RLGL–WE14 efficiently identified ChgA-reactive T cells in islets from prediabetic nonobese diabetic (NOD) mice. Finally, in the crystal structure of the IA^g7^–RLGL–WE14 complex, the peptide was bound in the IA^g7^ binding groove, as we predicted with RLGL in the p1 to p4 positions and the WSRMD of WE14 in p5 to p9 positions (illustrated in Fig. 1 A).

The super-agonist activity of the pS3 and the chimeric RLGL–WE14 peptide suggested that, when bound to IA^g7^, they create very high-affinity ligands. To test this idea, we prepared a soluble version of the BDC-2.5 TCR and used surface plasmon resonance (SPR) to examine its affinity for the two peptides bound to IA^g7^ (Fig. 1 B). These IA^g7^ complexes were immobilized in two flow cells of a BIAcore BIAsensor chip, while IA^g7^ bound to a negative control peptide from hen egg lysozyme (HEL) peptide was placed in a third flow cell. Various concentrations of the BDC-2.5 TCR were injected, and the association/dissociation kinetics were followed via the SPR signal (resonance units [RUs]). The figure shows the data for the two agonists after correction for the fluid-phase SPR signal using the data from the negative control flow cell. The TCR bound equally strongly to the two agonist IA^g7^ complexes with second-order kinetics. Following an initial rapid on rate (ka1), there were two off rates, one fast (kd1) and one slower (kd2), indicating that, during the bound time, some of the complex made a conformational change (ka2), prolonging its bound time. This led to an overall apparent dissociation constant of <1 µM, confirming the very high affinity of this TCR for these ligands.

Based on the properties of the p1 to p4 portion of the IA^g7^ binding groove and the predicted p1 to p4 amino acids of the stimulatory library peptides reported by us and others, we suggested a list of peptide fragments from other β cell granule proteins that, when fused to WE14, might create super-agonists similar to RLGL–WE14 (Jin et al., 2015). One of these peptides was a fragment of proinsulin C-peptide, TLAL. The activity of the TLAL–WE14 chimeric peptide and its presence in pancreatic islets and β cell tumors has now been demonstrated by others (Delong et al., 2016; Wiles et al., 2019). However, since our analysis suggested that fragments of other granule proteins could also complete the epitope, we used our baculovirus platform (Crawford et al., 2006; Stadinski et al., 2010a) to construct a library of peptides covalently bound to IA^g7^ expressed on the surface of infected SF9 insect cells. In this case, the library encoded the first five amino acids of WE14 (WSRMD) in the p5 to p9 positions, with an N-terminal extension that included randomized amino acids at the p1 to p4 positions of the encoded peptide. This library was large enough (∼10^7^) to contain virtually all combinations of amino acids at these positions.

SF9 cells were infected with a control IA^g7^–HEL-encoding virus or with virus encoding the unsorted IA^g7^ peptide library at a multiplicity of infection of ∼1. After 3 d, the infected cells were tested for IA^g7^ surface expression versus binding of a fluorescent multimeric version of the BDC-2.5 TCR. The control SF9 insect cells infected with an IA^g7^–HEL-expressing virus expressed the complex very well on the surface but failed to bind the TCR (Fig. 1 C, panel 1). Infected SF9 cells expressing IA^g7^ bound to the peptide library also expressed IA^g7^ well (Fig. 1 C, panel 2), but in this case, ∼3% of those cells also bound the BDC-2.5 TCR, suggesting that epitopes for BCD-2.5 were quite frequent, even in the unenriched library. The TCR-binding insect cells were sorted to make a new stock of enriched virus, which was expanded and then used for a second round of infection and analysis (Fig. 1 C, panel 3). This stock was highly enriched for functional peptides, since ∼50% of the infected cells that were expressing IA^g7^ now bound the TCR as well. Expanding the virus from the TCR-binding cells for another round of infection and analysis showed no further enrichment of TCR binding (Fig. 1 C, panel 4). Therefore, pooled baculovirus DNA was prepared from the TCR-binding SF9 cells in Fig. 1 C, panel 3, and was used as a template in a PCR to create a DNA fragment encoding the sequences of the enriched peptides. The fragment was sequenced and yielded ∼200,000 functional, in-frame sequences encoding ∼10,000 unique peptide sequences occurring between 1 and ~13,000 times in the sample. Eliminating those occurring <10 times left ∼165,000 total sequences encoding 2,260 unique sequences (Data S1), which were used to create a WebLogo amino acid frequency plot (Crooks et al., 2004) representing the BDC-2.5 TCR-selected motif for p1 to p4 positions of the active peptides in IA^g7^ (Fig. 1 D).

The motif showed that the p1 anchor position selected many different amino acids, consistent with the amphipathic nature of the p1 pocket of IAg7 (Suri et al., 2005). Many different amino acids were also found at the surface-exposed p2 position, but, except for R, amino acids with aliphatic or small neutral side chains were favored. At the surface-exposed p3 position, P was the amino acid most heavily selected by the BDC-2.5 TCR, followed by the small neutral amino acids, S, G, T, and A. Aliphatic amino acids, especially L, were selected at the p4 anchor position. These results agree very well with previous studies on the properties of peptides found bound to IA^g7^ (Suri et al., 2005). Our RLGL–WE14 and the TLAL–WE14 agonists also match the motif, as do many of the peptide library agonists for BDC-2.5 reported by others (Judkowski et al., 2001; Yoshida et al., 2002). Among these previous library peptides, those with a p2R and/or p3P often stimulated BDC-2.5 very well, but not other ChgA-specific T cells with unrelated TCRs, suggesting that among the peptides selected by the BDC 2.5 TCR from our library, some would be uniquely specific for BDC-2.5.

**Figure 2.**
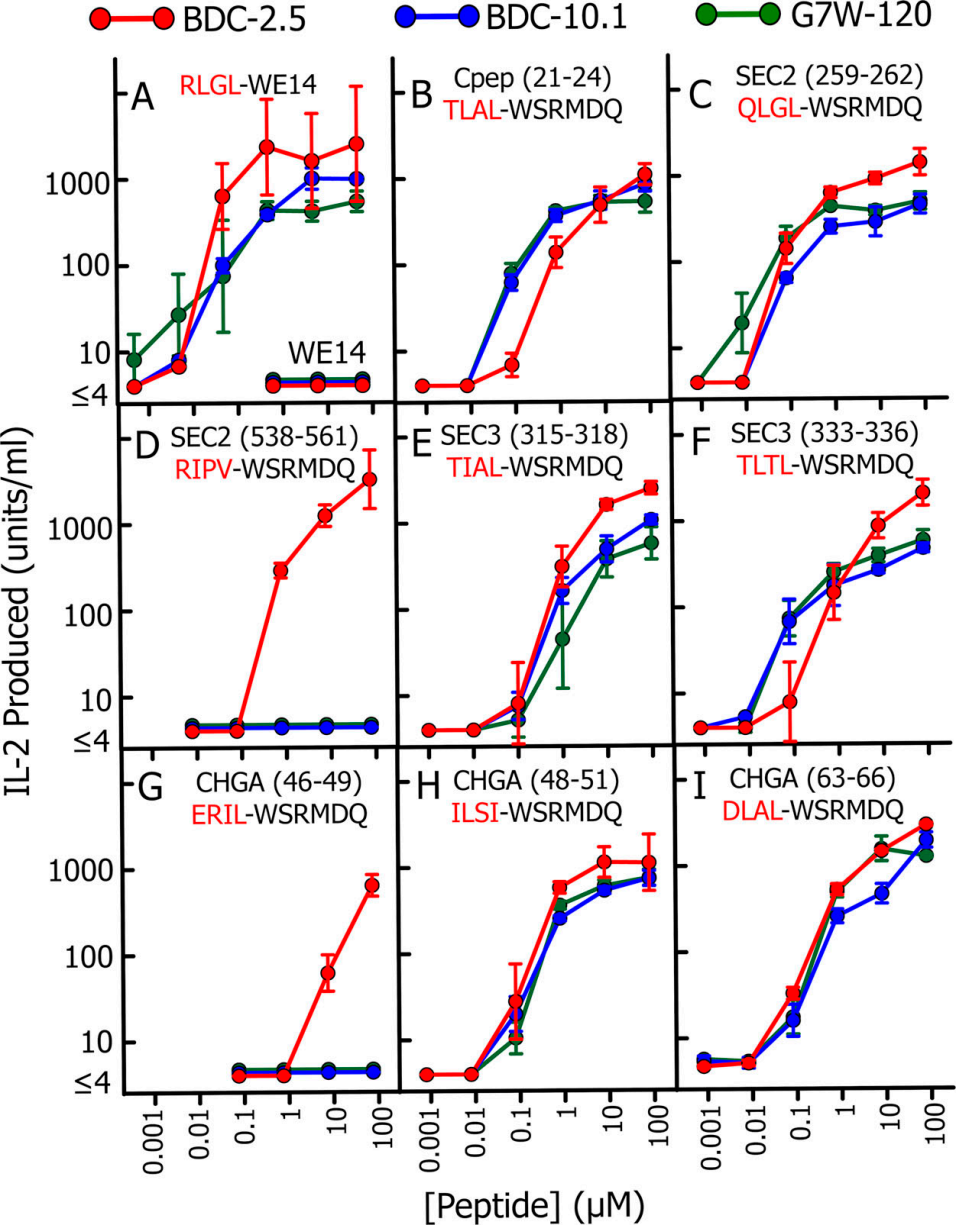
**Multiple prohormone-derived peptides, when synthetically fused to the Nterminus of WE14, create super-agonists for WE14-specific T cells.** **(A–I) **Various peptides were titrated with paraformaldehyde-fixed M12.C3.g7 APCs in IL-2 production stimulation assays with three WE14-specific T cells: BDC-2.5 (red), BDC-10.1 (blue), and G7W-120 (green). The results are the average ± SEM of IL-2 produced in three separate experiments. The peptides were RLGL–WE14 and unmodified WE14 (A); C-peptide (21–24), TLAL, fused to WSRMDQ (B); Sec2 (259–262), QLGL, and (538–561), RIPV, fused to WSRMDQ (C and D); Sec3 (315–318), TIAL, and (333–336), TLTL, fused to WSRMDQ (E and F); ChgA (46–49), ERIL, (48–51), ILSI, and (63–66), DLAL, fused to WSRMDQ (G–I). Data shown are the average of three experiments with the SEM.

**Figure S1.**
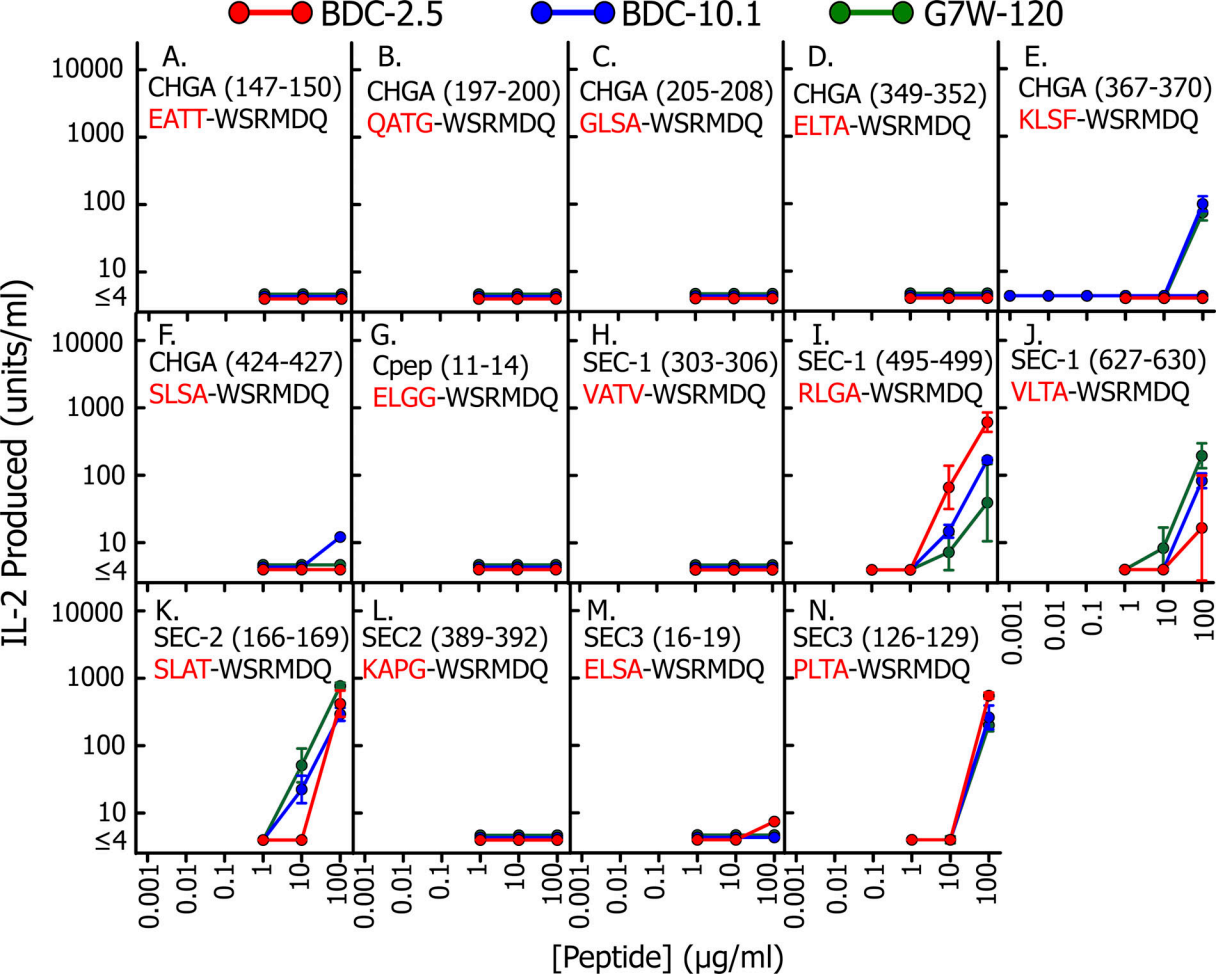
**Four–amino acid peptides that, when fused to WSRMDQ fragment of WE14, have weak or no activity in stimulating WE14-specific T cells.** This figure supplements Fig. 2. Among the 22 four–amino acid peptides fused to WSRMDQ that matched to some extent the peptide motif described in Fig. 1 D, 8 showed strong stimulation of the WE14-specific T cells BDC-2.5, BDC-10.1, and G7W-120 or just BDC-2.5. Their activities are presented in Fig. 2. This figure shows the data for the other 14 peptides that, when tested, had either weak or no activity with these T cells. Details of the experiments are in Materials and methods and the figure legend to Fig. 2. All assays were performed three times, and the averages ± SEM are shown.

We used our previous data and those in Fig. 1 D to select a set of 20 tetrapeptides from abundant β cell granule proteins, C-peptide (including TLAL), secretogranin 1 (Sec1), Sec2, Sec3, and ChgA itself that were consistent with the p1 to p4 motif. Each of these tetrapeptides was synthesized as a fusion to the N terminus of a WE14 fragment, WSRMDQ. The peptides were titrated for their ability to stimulate three different WE14-reactive CD4 T cells, BDC-2.5, BDC-10.1, and G7W-120, a BDC-10.1–related T cell (Jin et al., 2015), using IA^g7^ APCs. Control peptides were our RLGL–WE14 agonist and the unmodified WE14 peptide (Fig. 2 A). The results with the eight most stimulatory hybrid peptides are shown in Fig. 2, B–G. The results with the other 12 peptides, which showed poor or no activity, are shown in Fig. S1. As we have previously shown (Jin et al., 2015), the RLGL–WE14 peptide stimulated all three T cells very well, but the unmodified WE14 peptide was inactive at the concentrations tested here (Fig. 2 A). Six of the eight hybrid peptides in the other panels of Fig. 2, including the TLAL–WE14 peptide, strongly stimulated all three T cells (Fig. 2, B, C, E, F, H, and I), while the other two (Fig. 2, D and G) stimulated only BDC-2.5. Notably, the BDC-2.5–specific peptides contained a p2R or p3P, similar to the highly stimulatory, previously reported BDC-2.5–specific library peptides mentioned above. All of these peptides had an optimal anchor, L, I, or V, at p4. In contrast, all but one of the poorly stimulating peptides in Fig. S1 lacked one of these optimal p4 anchor amino acids.

### Creation of super-agonists by treatment of a WE14 donor and proinsulin C-peptide acceptors with Cat-L

**Figure S2.**
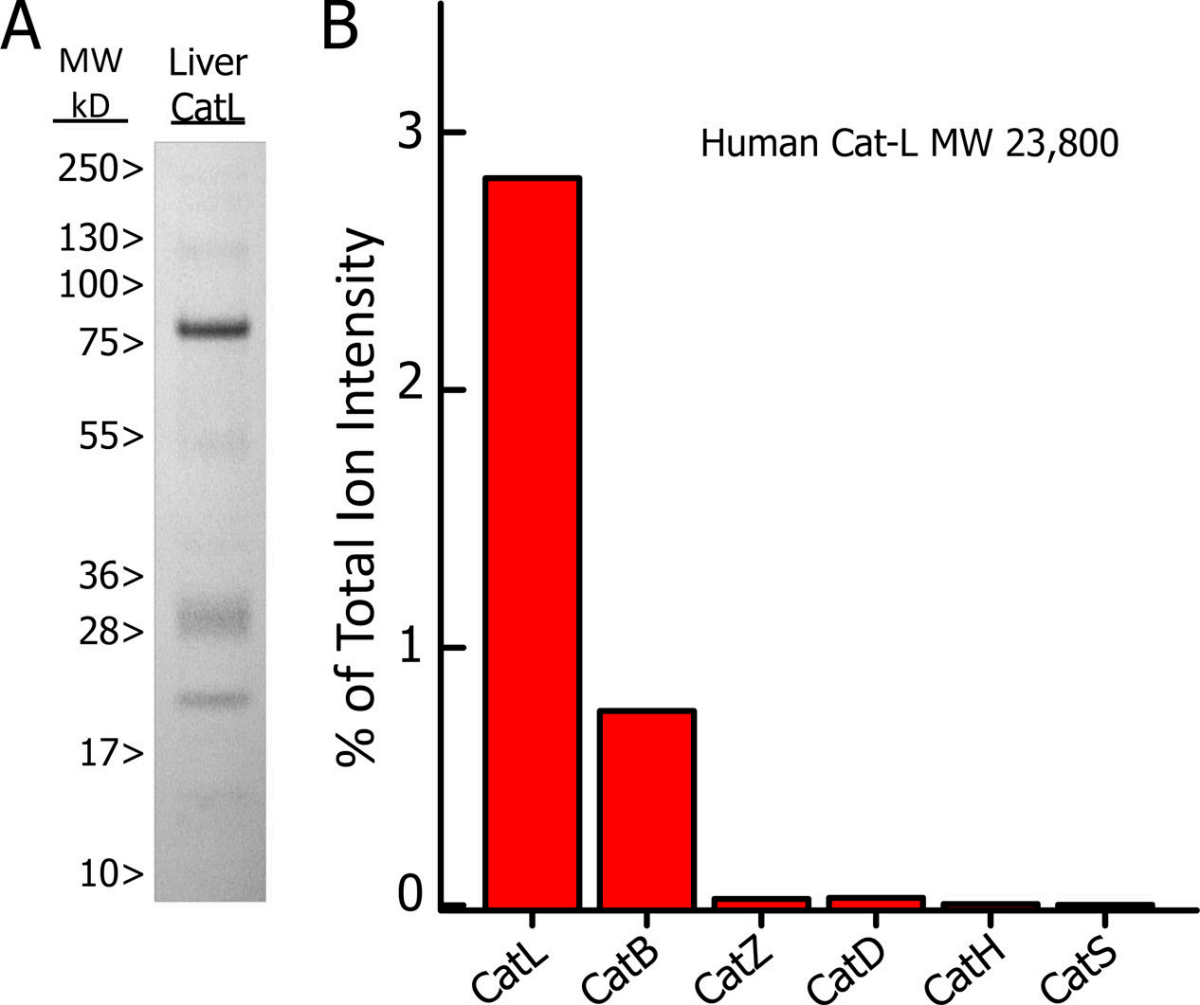
**Commercial human livCat-L is heavily contaminated with other proteins, including several cathepsins.** Data documenting the presence of many proteins contaminating the commercial livCat-L, among which were several cathepsins. **(A)** 3.4 µg of a commercial human livCat-L was run on a 4–12.5% NuPAGE SDS-PAGE Bis-Tris gel (NP0336; Invitrogen) under reducing conditions, and the gel was stained with Coomassie Blue. Prestained mol wt (MW) markers (Thermo Fisher Scientific), run in a parallel lane, are shown. **(B)** The protein was eluted from the gel and digested with trypsin. The digest was subjected to MS/MS analysis against a library of human proteins. Among the peptides detected were those from Cat-L and other cathepsins. The sum of the ion intensities of the tryptic fragments from each cathepsin are plotted as the percentage of the total ion intensities for the tryptic fragments for all of the proteins detected in the sample.

These results encouraged us to test whether some of these active chimeric peptides could be generated in vitro via a transpeptidation reaction mediated by lysosomal cathepsin proteases. First, we began with C-peptide that contained TLAL, which could generate a compatible acceptor for fusion to a WE14 donor if cleaved after the second L in TLAL. Several previous studies had demonstrated the presence of fragments of C-peptide truncated at the C terminus of TLAL in rat and mouse islet cell preparations (Boonen et al., 2007; Delong et al., 2016; Verchere et al., 1996), and this chimeric TLAL–WE14 peptide had been previously identified (Delong et al., 2016). In preliminary experiments, we digested intact human proinsulin with two human cathepsins isolated from liver (livCat-L and cathepsin B [livCat-B]) and one recombinant (cathepsin S [recCat-S]) expressed in *Escherichia coli*. The digests were analyzed by MS/MS to identify the N termini of the cleavage products (Data S2). Only the livCat-L cleaved the C-peptide after PLAL (homologous to the TLAL in mouse C-peptide). Therefore, we examined the cleavage of free mouse C-peptide with the livCat. We subsequently found that the commercial livCat-L we were using was only ∼3% Cat-L and was significantly contaminated with other proteins, including human Cat-B (∼0.75%; Fig. S2), so we obtained a purified (>95%) recombinant Cat-L produced in *E. coli* (recCat-L) and repeated the digestion of mouse C-peptide. The cleavage after TLAL was found in both digests (Data S2).

**Figure 3.**
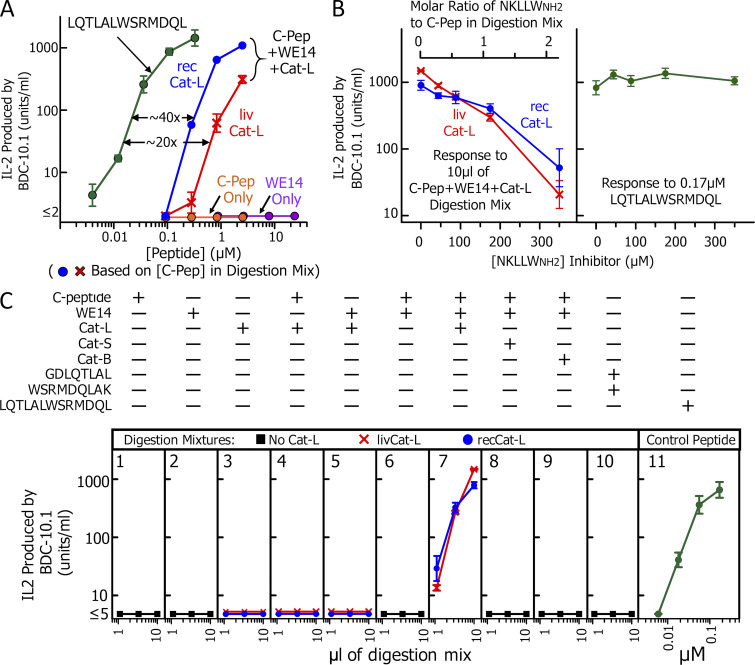
**Cat-L creates an agonist for BDC-10.1 from an inactive mixture of C-peptide and WE14.** A complete general protocol for the transpeptidation digestion reactions we performed can be found in Materials and methods. **(A)** Digestions were performed in 20 µl containing 10 µg of mouse INS-1 C peptide (mol wt 3,122) and 50 µg of WSRMDQLAKELTAE (mol wt 1,678), with an estimated 10 ng (assuming 3% purity) of human livCat-L (red ×) or 12.5 ng of recCat-L (blue circle). Various volumes of the pH-neutralized digestion mixes were tested for stimulation, as measured by IL-2 production, of the BDC-10.1 WE14-specific T cell using IA^g7^-expressing M12.C3.g7 cells as APCs. The results are plotted as IL-2 produced versus the molar concentration of C-peptide (before digestion) in the digestion mix. Control stimulations are shown with undigested C-peptide (orange circle) and WE14 (purple circle) and the synthetic control chimeric peptide, LQTLALWSRMDQL (green circle). **(B)** Left: Transpeptidation digestions were performed with both Cat-Ls as in A in the presence or absence of increasing concentrations (44-350 µM) of a competitive Cat-L inhibitor, NKLLW_NH2_. The plot shows the response of BDC-10.1 T cells to 10 µl of the neutralized digestion mixtures versus the concentration of the inhibitor in the digestion. The scale at the top shows the molar ratio of the inhibitor to C-peptide in the digestions. Right: The response of BDC-10.1 to the control LQTLALWSRMDQL peptide (0.17 µM) in the presence or absence of the amount of NKLLW_NH2_ that would be carried over from the stimulation assays shown in the left panel. **(C)** The components of various digestion mixes are shown above each panel with their stimulation results with BDC-10.1. Panels 1–7: All possible combinations of no peptide, C-peptide, and WE14 with either no Cat-L (black square), livCat-L (red ×), or recCat-L (blue circle) were incubated under digestion conditions, and then 1.1, 3.3, and 10 µl of the neutralized reaction mix were tested for stimulatory activity for BDC-10.1. Panels 8 and 9: As other panels, but with 30 ng of recCat-S or 22 ng of livCat-B, rather than Cat-L. Panel 10: Same as panel 7 except the mix contained only 2.7 µg of the C-peptide fragment GDLQTLAL and 34 µg of the WE14 fragment WSRMDQLAK, but no Cat-L. Panel 11: Control response to the synthetic chimeric LQTLALWSRMDQL peptide. All results in this figure are the average of three experiments ± SEM.

With this information, we digested a mixture of mouse C-peptide and mouse WE14 with the livCat-L or recCat-L under lysosomal conditions and then tested the digests for their ability to stimulate IL-2 production by the BDC-10.1 T cell, using the IA^g7^-bearing cell line, M12.C3.g7, as the APC. As a positive control, we used a synthetic peptide, LQTLALWSRMDQL, containing the preformed chimeric epitope (p1 to p9, underlined). As shown in Fig. 3 A, the control peptide was highly stimulatory, but neither C-peptide nor WE14 alone had any activity at the concentrations used in the digests. However, both Cat-L digests contained a stimulatory peptide. The results for digestion mixes were plotted in this figure based on concentration of C-peptide added to the mix. By comparison of the responses to the control peptide versus the digestion mix, we estimated that ∼2.5–5% of the C-peptide was converted to a stimulatory peptide by the digestion.

We repeated the digestions with several controls to rule out other interpretations of our results. To establish that Cat-L, but not another contaminating protease, generated the activity, we performed the digestions in the presence of various concentrations of the Cat-L–specific competitive inhibitor, RKLLW-NH_2_ (Brinker et al., 2000), and tested 10 µl of each digest for the ability to stimulate BDC-10.1 (Fig. 3 B). The results show that the generation of the active peptide by either Cat-L was inhibited by RKLLW-NH_2_ in a dose-dependent manner, with ∼50% inhibition when the inhibitor was at the same concentration as the C-peptide substrate in the digestion (Fig. 3 B, left). As a control for nonspecific inhibition of the stimulation assay by RKLLW-NH_2_ carried over from the digestion, we prepared media with these same concentrations of RKLLW-NH_2_ but lacking the acceptor and donor peptides and Cat-L. These preparations were not inhibitory when tested in the response of BDC-10.1 to a limiting amount (0.17 µM) of the synthetic chimeric control peptide (Fig. 3 B, right).

We also tested various combinations of input peptides and cathepsins, and the results are shown in Fig. 3 C, panels 1–11. The first seven panels show that, for both enzymes, no combination of C-peptide, WE14, and Cat-L (Fig. 3 C, panels 1–6), except the mixture of all three (Fig. 3 C, panel 7), generated the active product. Fig. 3 C, panels 8 and 9, show that, as predicted, recCat-S and livCat-B could not replace Cat-L in the digestion. In Fig. 3 C, panel 10, we ruled out the possibility that the Cat-L digestion was required simply to generate a C-peptide fragment ending in TLAL, but that this unfused fragment could synergize with WE14 to fill the IA^g7^ binding groove. Thus, a mix of an N-terminal WE14 fragment and a synthesized fragment of C-peptide already ending in TLAL did not stimulate BDC-10.1 at the concentrations used in our digests. Fig. 3 C, panel 11, shows again the stimulatory activity of synthetic peptide bearing the prefused chimeric epitope.

### Other acceptors for WE14 generated by Cat-L cleavage of other proteins

Based on the data in Fig. 2, we predicted that various islet granule proteins, other than C-peptide, might also be cleaved by Cat-L to form an appropriate acceptor for WE14 transpeptidation. To test this idea, we used five 12–amino acid fragments of ChgA, Sec3, and as a control, C-peptide, each containing internally the four amino acids required for fusion to the WE14 N terminus to generate the active chimeric epitope. Each of these peptides was mixed with an N-terminal fragment of WE14, WSRMDQLAK, and digested with livCat-L or recCat-L. We used these shorter versions of the peptides in these digestions to simplify the MS/MS analysis by limiting the masses of the potential chimeric peptides generated, allowing them to fall within the optimal range for the instrument without the need for further digestion. Part of each digestion mix was tested for stimulation of BDC-2.5 and BDC-10.1. The remainder of the mix was reserved for later MS/MS analysis.

**Figure 4.**
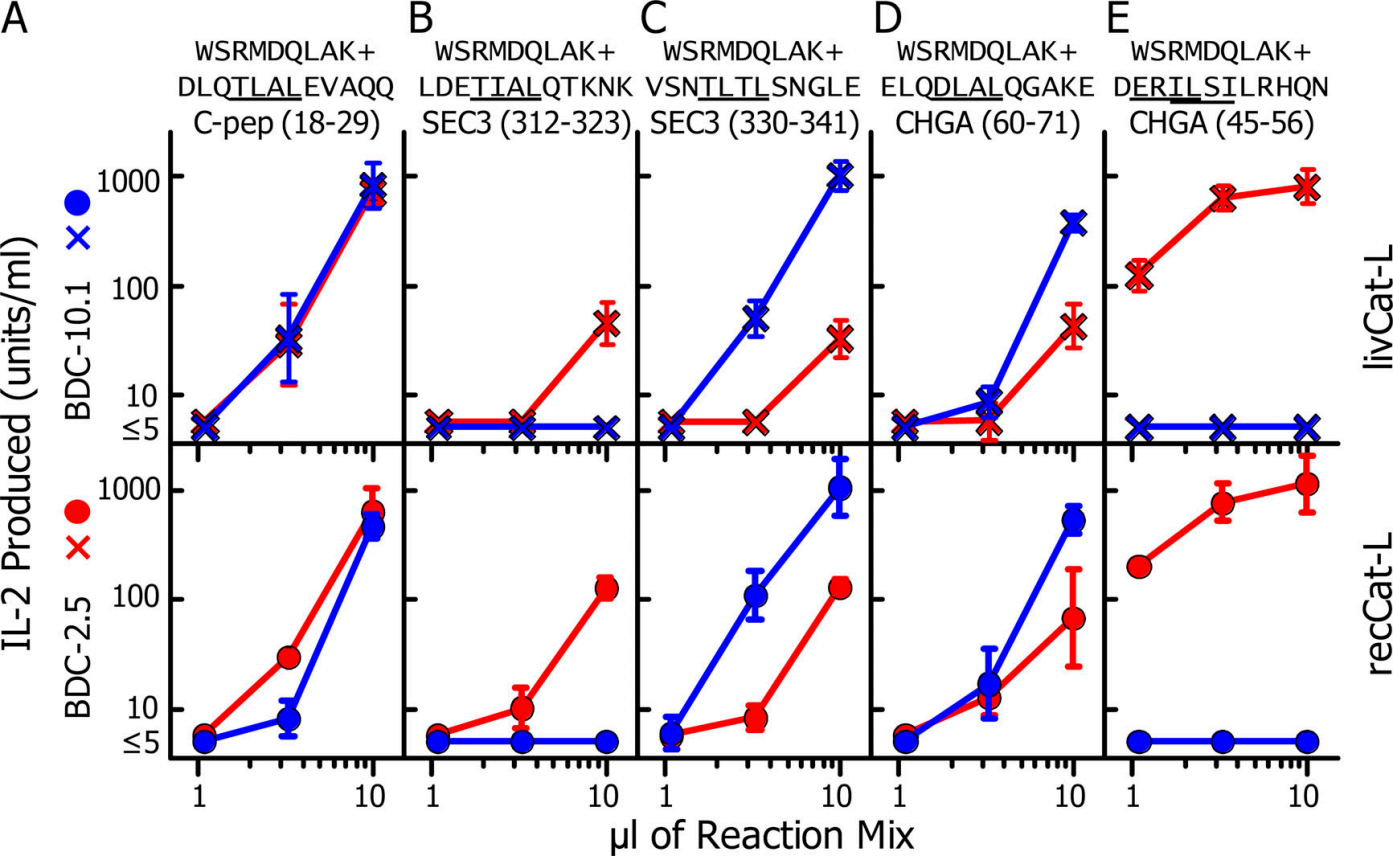
**Successful Cat-L generation of agonists for BDC-10.1 and BDC-2.5 from mixtures of fragments of WE14 and several other granule proteins. (A–E)** Digestions were performed using both livCat-L and recCat-L, with the fragments from the granule proteins C-peptide, Sec3, and ChgA shown at the top of each figure as acceptors, and the WE14 fragment, WSRMDQLAK, as donor. The acceptor:donor ratios in the 10-µl digestion reactions were 1:7 µg/µl (A) and 3:9 µg/µl (B–E). The digestions were performed with either 10 ng of livCat-L or 12.5 ng of recCat-L. Three volumes of the neutralized digestion reaction (1.1, 3.3, and 10 µl) were tested for stimulation of BDC-10.1 (blue) and BCD-2.5 (red) T cells using M12.C3.g7 cells as APCs. The results with the livCat-L digests are shown in the upper half of the figures (×) and to the recCat-L in the lower half (solid circle). All results are the average of three experiments ± SEM.

Fig. 4, A–E, shows the stimulation data. The sequence of the input WE14 fragment and five peptides containing the potential acceptors are at the top of each figure, with the minimal four–amino-acid acceptors underlined. In Fig. 4 E, two sequences are underlined, since the data in Fig. 2, G and H, indicated that two potential acceptor sites are present. The stimulation results are shown below the sequences, with the results with livCat-L digests above and recCat-L below. The two Cat-L preparations yielded virtually identical results, indicating that the results with livCat-L were independent of its contaminants (see Materials and methods). As with the full-length C-peptide, the digestions with the C-peptide fragment were active in stimulating both BDC-2.5 and BDC-10.1 (Fig. 4 A). The other digestion mixes stimulated BDC-10.1 and/or BDC-2.5 with variable potency. Notably, the digest assayed in Fig. 4 E stimulated BDC-2.5 very strongly, but BDC-10.1 not at all, suggesting that the ERIL acceptor dominated over the ISLI acceptor.

### MS/MS analysis reveals the presence of the predicted chimeric peptides in the Cat-L digests

**Figure 5.**
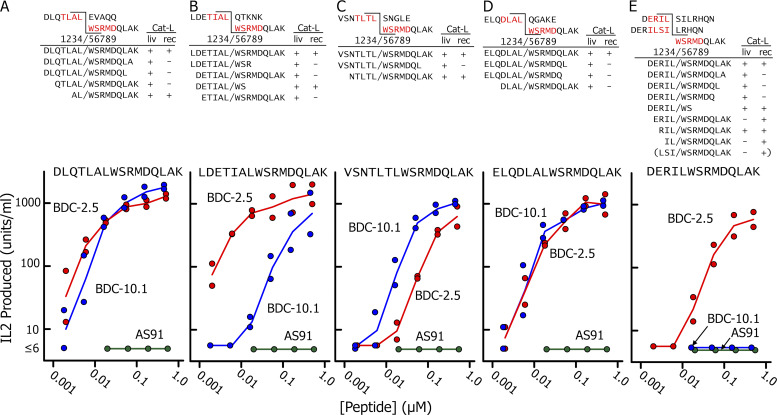
**Identification and activity validation of the predicted chimeric peptides in the Cat-L digests. (A–E)**. The upper portion of each figure shows the sequences of the input acceptor and donor peptide for each digestion in Fig. 4 with the predicted transpeptidation-generated fusions indicated that would generate a chimeric functional epitope. The minimal nine–amino acid functional epitope is highlighted in red, and the predicted positions of the amino acids in the IA^g7^ binding groove are numbered. The digests were analyzed by MS/MS, and all of the peptides identified containing this fusion are listed, along with which Cat-L digest contained them. The lower portion of each figure shows the responses of three T cells, BDC-10.1 (blue circle), BCD-2.5 and (red circle), and AS91 (green circle), to a synthetic version of the full-length chimeric peptide, which was identified in each case and whose sequence is shown at the top of each panel. The stimulations were performed twice, and the results of both experiments are shown.

**Figure 6.**
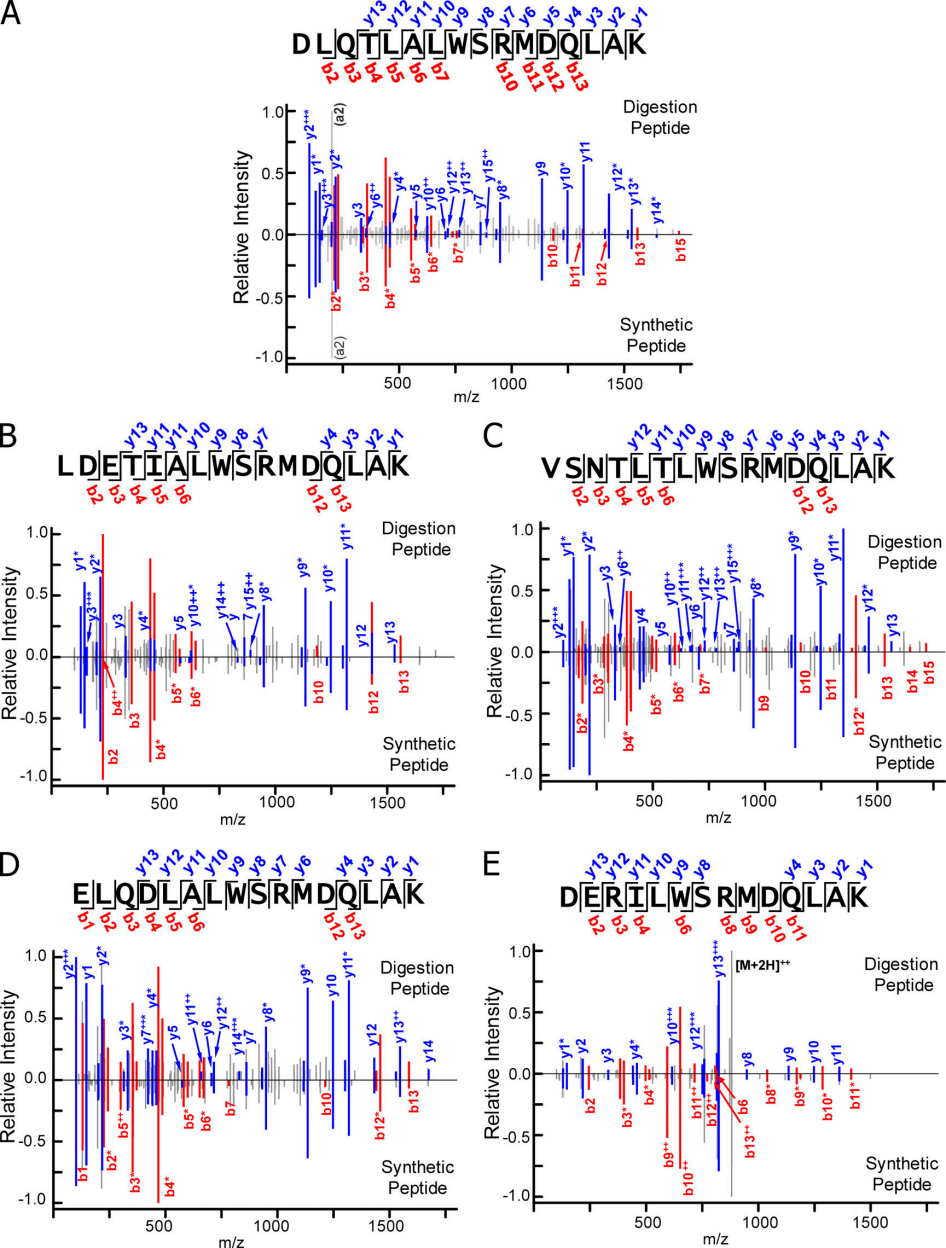
**Comparison of the MS/MS fragmentation patterns of chimeric peptides in the digests to synthetic versions confirm their identifications. (A–E)** Each figure displays the MS/MS fragmentation patterns obtained with the longest functional chimeric peptide found in the recCat-L digests in Fig. 5 (the data for the livCat-L is shown in Fig. S3). The relative ion intensity values for all identified singly or doubly charged y (blue) and b (red) ion fragments normalized to the intensity of most intense fragment found in the sample (set to 1.0) are plotted along the x axis at the m/z value for the fragment. Some fragments had neutral variants that had lost NH_3_ or H_2_O. For a cluster containing the loss variants alone or in combination with the intact fragment, the y or b labels are marked with an asterisk. Other fragments present in the spectra are colored gray. These include a-ions (not labeled unless used for normalization), unidentified fragments (also unlabeled), and the unfragmented full-length peptide ([M+2H]^2+^). For validation, the MS/MS fragmentation pattern obtained on the same instrument with a synthetic version of the same chimeric peptide is plotted along the same x axis but in the negative direction. Within the sequence of the peptide at the top of each figure, the y and b ions are bracketed when they and/or their derivatives were present in both spectra.

For each digestion in Fig. 4, we created a database of several hundred peptides (Data S3) that contained all possible fusions between the input peptides in any direction, such that in resulting chimeric peptides, the N terminus of the acceptor and the C terminus of the donor had not been truncated. This database contained the full-length chimeric peptide we predicted as the functional one in our stimulations in Fig. 4 but also hundreds of other full-length chimeric peptides. As described in Materials and methods, the digests in Fig. 4 were analyzed using MS/MS, matching the data against our databases looking for spectral data that matched any portion of the theoretical peptides. Chimeric peptides contained the fusion site in the matching peptide. They could include peptides that were truncations of the full-length database peptide from either end. Those not containing the fusion site were fragments of the input peptides. Data S4 lists all of the peptides identified with these databases. The first two sheets in Data S4 list the chimeric peptides in which the N-terminal W of input WSRMDDLAK peptide as a donor was found fused to any position in the other input acceptor peptide. The data from the livCat-L and recCat-L digestions are in separate sheets. These data were used to construct the plots in Figs. 5 and 6 and the first part of Fig. 8.

At the top of Fig. 5, A–E, we show the predicted functional fusions between the input acceptors and the WE14 fragment in each digestion that would generate the longest possible chimeric peptide. Chimeric peptides identified in the digests having this predicted fusion site are listed below in the figures. All of the digestions with either version of Cat-L contained this full-length fusion peptide, and these were generally the most abundant peptide in each set (Data S4). Versions of this peptide with truncations at either end were also found, usually with lower ion intensities, suggesting that the initial fusion event occurred between the original input peptides, with these shorter peptides generated by trimming of the ends. Most, but not all, of the peptides contained the nine–amino acid minimal epitope, four amino acids from the acceptor and WSRMD from the WE14 peptide. In a few cases, the truncations had removed part of this epitope, generating a peptide predicted to have less or no activity. Fig. 5 E shows that many versions of the fusion of the ChgA ERIL peptide occurred with both Cat-L proteases, but only a single peptide was identified (in the recCat-L digest) that had the fusion to ILSI (in parentheses in Fig. 5 E). This peptide was truncated, missing the initial p1 amino acid of the epitope, which presumably accounted for the lack of activity of the digest with BDC-10.1.

As an additional control, the lower portions of Fig. 5, A–E, show the activities of synthetic versions of the full-length chimeric peptides found in the digests in stimulating BDC-2.5, BDC-10.1, and the negative control insulin-specific T cell, AS91. As predicted, none of the peptides stimulated AS91, but their relative potencies in stimulating BDC-2.5 versus BDC-10.1 closely matched those seen with the original digestion mixes in Fig. 4. Thus, the TLAL- and DLAL-containing peptides did not distinguish the two T cells (Fig. 5, A and D), while the TIAL-containing peptide favored BDC-2.5 (Fig. 5 B) and the TLTL-containing peptide favored BDC-10.1 (Fig. 5 C). Also, as before, the ERIL-containing peptide stimulated only BDC-2.5 (Fig. 5 E), likely explained by the inhibitory R at p3 for the BDC-10.1 response discussed above. These data indicate that the MS/MS-identified peptides were in fact the peptides responsible for the activities in the digests. It is worth pointing out that these longer synthetic peptides were, in general, more active than corresponding peptides used in Fig. 2, which were synthesized with only the p1 to p4 acceptor amino acids fused to the WE14 fragment donor. The effect of the additional amino acids was most dramatic in the responses of BDC-2.5 to the TIAL- and ERIL-containing epitopes, whose responses were improved ∼100× by the additions. Likely, these differences were mostly due to the added p1 amino acid, whose side chain has often been shown to interact with the T cell receptor in crystal structures of TCR–MHCII–peptide complexes. (e.g., Wang et al., 2019).

**Figure S3.**
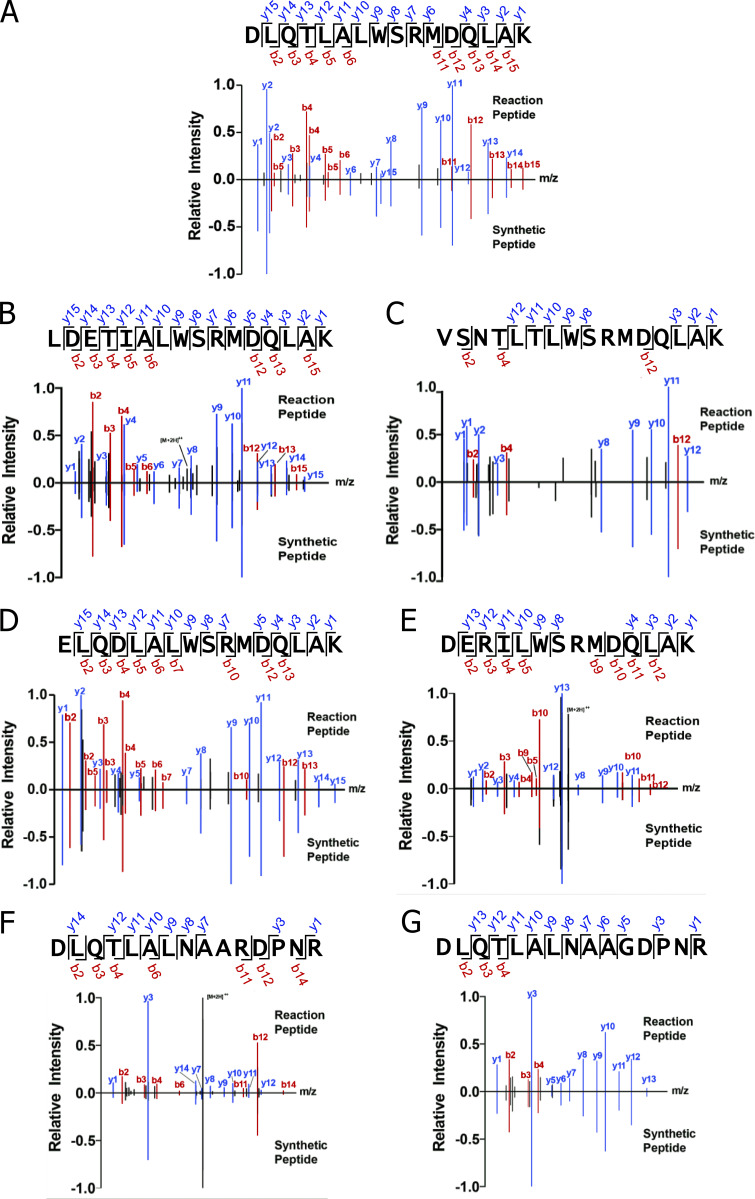
**MS/MS fragmentation patterns for the seven full-length functional chimeric peptides generated with livCat-L match those for synthetic versions of the same peptides.** The seven transpeptidation reactions described in Figs. 4 and 7 were performed separately with livCat-L and recCat-L. The full-length functional chimeric epitopes were identified in all 14 reactions. The intensities of the detected y and b fragments generated from this peptide during MS/MS analysis were plotted versus their m/z values and compared with those generated from a synthetic version of the same peptide. The results of with recCat-L digestions are shown in Figs. 6 and 7. This figure shows the results of the analysis of the livCat-L digestions performed with a different instrument. The data are presented similarly to those in Figs. 4 and 7. See Materials and methods and the figure legends for Figs. 6 and 7 for more details. **(A–E)** Peptides generated with various acceptors and the WE14 fragment WSRMDQLAK as donor. **(F and G)** Peptides generated with the C peptide fragment DLQTLALEVAQQ as acceptor and the NAARDPNR or NAAGDPNR proIAPP peptide fragments as donors.

As a final check that the MS/MS analysis correctly identified the sequences of the functional chimeric peptides in the digests, we compared the MS/MS fragmentation spectra attributed to the functional full-length fused peptides in our Cat-L digestions to the spectra obtained with synthetic versions of the same peptides tested in Fig. 5. In both cases, the y and b fragments were identified in the MS/MS spectra using a database of all potential y and b ions for each peptide (Data S5). A summary of the data for the recCat-L is shown in Fig. 6, A–E. The data for the livCat-L are shown in Fig. S3, A–E. The relative ion intensities of the y (blue) and b (red) fragments are plotted in each figure against the m/z value of the fragment, with the data for the digestion peptide in the positive direction and for the synthetic peptide mirrored in the negative direction. The cleavage points for y and b fragments that were identified in both spectra are bracketed on the sequence of the peptide at the top of each figure. The major y and b fragments in all the comparisons were nearly identical, with similar relative intensities confirming the identification of the chimeric epitope peptides in the digests.

### Cat-L also creates an active chimeric peptide for an IAPP-reactive T cell

**Figure 7.**
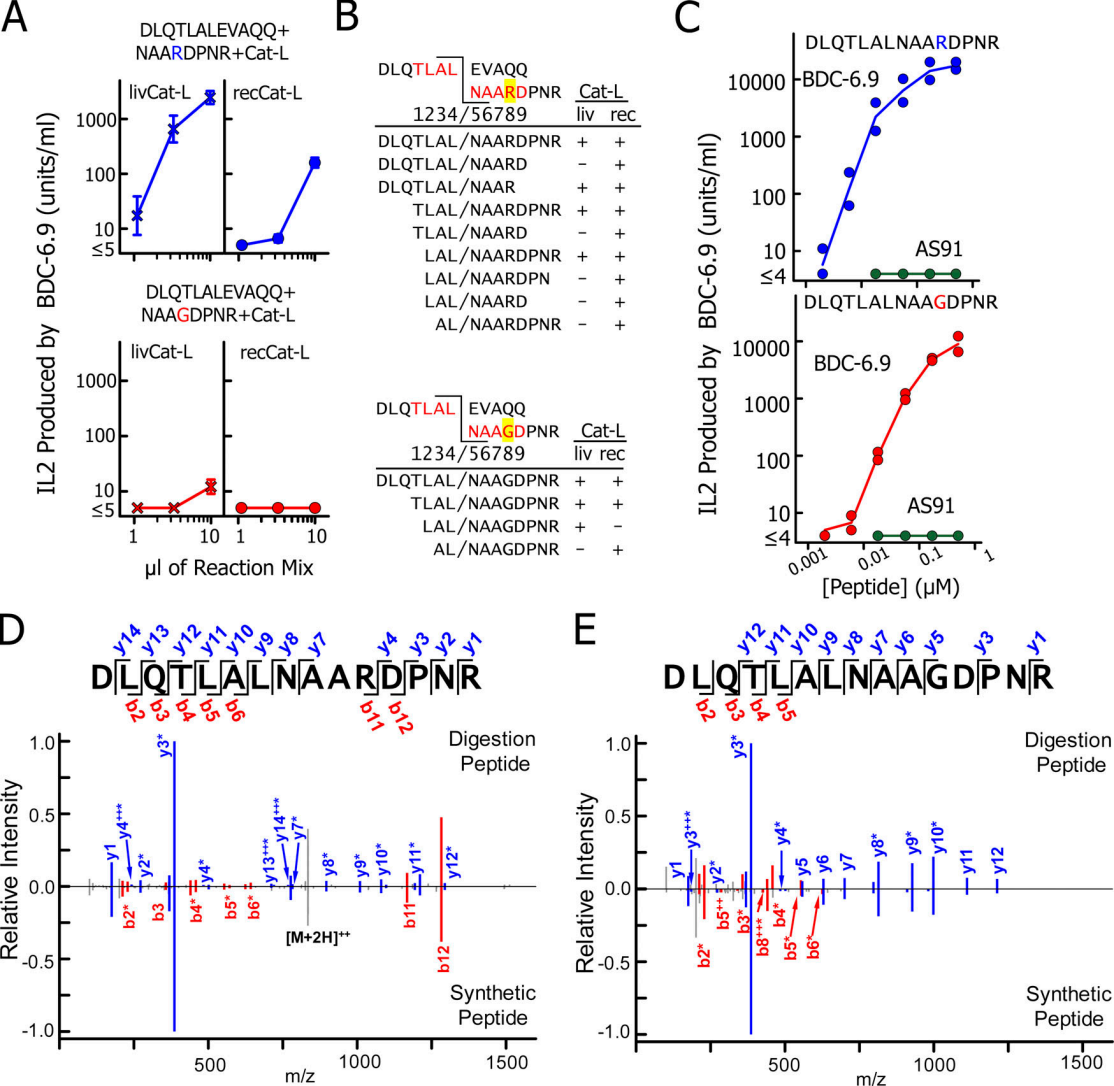
**Cat-L can also create super-agonist for the IAPP-specific T cell BDC-6.9. (A)** Digestions with either 10 ng of livCat-L (left) or 12.5 ng of recCat-L (right) were performed as in Fig. 4, using a mixture of the C-peptide fragment DLQTLALEVAQQ (0.5 µg/µl) and an IAPP fragment from either NOD mouse proIAPP, NAARDPNR (3.5 µg/µl; above), or BALB/c mouse proIAPP, NAAGDPNR (3.0 µg/µl; below). The neutralized digests were tested for stimulation of the IAPP-specific T cell, BDC-6.9, using the M12.C3.g7 APC as in Fig. 4. The digestions and stimulations were performed three times, and the results shown are the average ± SEM. **(B)** The predicted functional chimeric peptides in the digestions in A are shown with the peptides containing the fusions found by MS/MS listed below as in Fig. 5, with the NOD peptide data above that and with BALB/c peptide below. **(C)** Synthetic versions of the full-length chimeric peptides were tested for stimulation of the BDC-6.9 T cells and the negative control AS91 T cells as in Fig. 5. Each stimulation was performed twice, and both results are shown. **(D and E)** Comparisons of the MS/MS fragmentation pattern of the full-length chimeric peptide identified in the recCat-L digests shown in B versus that obtained with a synthetic version of the same peptide as presented in Fig. 6. Results with the NOD IAPP peptide are in D, and with the BALB/c peptide in E (similar results obtained with livCat-L are shown in Fig. S3).

The same acceptor C-peptide fragment ending in TLAL that, when fused to a WE14 donor, creates a highly stimulatory epitope for WE14-specific T cells, can also be fused to a fragment of the IAPP prohormone, creating a strong epitope for an IAPP-specific T cell, BDC-6.9, identified many years ago (Haskins et al., 1989; Wiles et al., 2017). In this case, the donor is the peptide released from the C-terminal end of the NOD proIAPP during its normal processing by prohormone convertase. Because of a polymorphism in this peptide, the equivalent peptide from BALB/c proIAPP, and other strains of mice lacking this polymorphism, is less stimulatory (Dallas-Pedretti et al., 1995). To test whether the functional IAPP chimeric epitope could be generated by transpeptidation, we performed a digestion as described as in Fig. 4 with both Cat-L preparations, using the same TLAL-containing C-peptide fragment, DLQTLALEVAQQ, as the acceptor. As donors, we used two versions of a proIAPP peptide fragment, one from NOD proIAPP, NAARDPNR, and one BALB.c proIAPP, NAAGDPNR, differing at the single underlined amino acid, predicted to be in the TCR interaction p8 position when bound to IA^g7^. The four digestions were performed and analyzed as in Figs. 4, 5, and 6, and the results are presented in Fig. 7.

The digests were tested for their ability to simulate IL-2 production by the NOD BDC-6.9 T cell (Fig. 7 A). The digests using the NOD donor peptide were stimulatory for BDC-6.9, but as expected, the digests using the equivalent BALB/c peptide were barely active at these concentrations. As above, the digests were analyzed by MS/MS using the databases in Data S3 containing all of the possible chimeric peptides that could be formed between the donor and acceptor peptides. The complete results of these analyses are listed in Data S4. As in Fig. 5, Fig. 7 B shows the sequences of the input peptides and the predicted fusion sites for the functional peptides and all peptides containing the predicted fusion in the two digests with either Cat-L. As in the digestions with the WE14 fragment as donor, the predicted full-length chimeric peptide was identified in each digest, along with various versions of this peptide truncated at either end. Again, this result suggested that the full-length fusion occurred first and that the other peptides were produced by subsequent trimming of this peptide. As in Fig. 5, synthetic versions of the two full-length fused peptides stimulated BDC-6.9, but the peptide containing the NOD donor was approximately five times more active than the peptide with the BALB/c donor (Fig. 7 C), accounting for the poor activity in the digestion with the BALB/c donor peptide. As with the WE14 fusions, comparison of the MS/MS fragmentation patterns of the full-length chimeric peptides identified in the digestions to those obtained with synthetic versions of the peptide confirmed their identification in the digests (Fig. 7, D and E).

### The many other fused chimeric peptides in the digests are consistent with the cleavage properties of Cat-L

**Figure 8.**
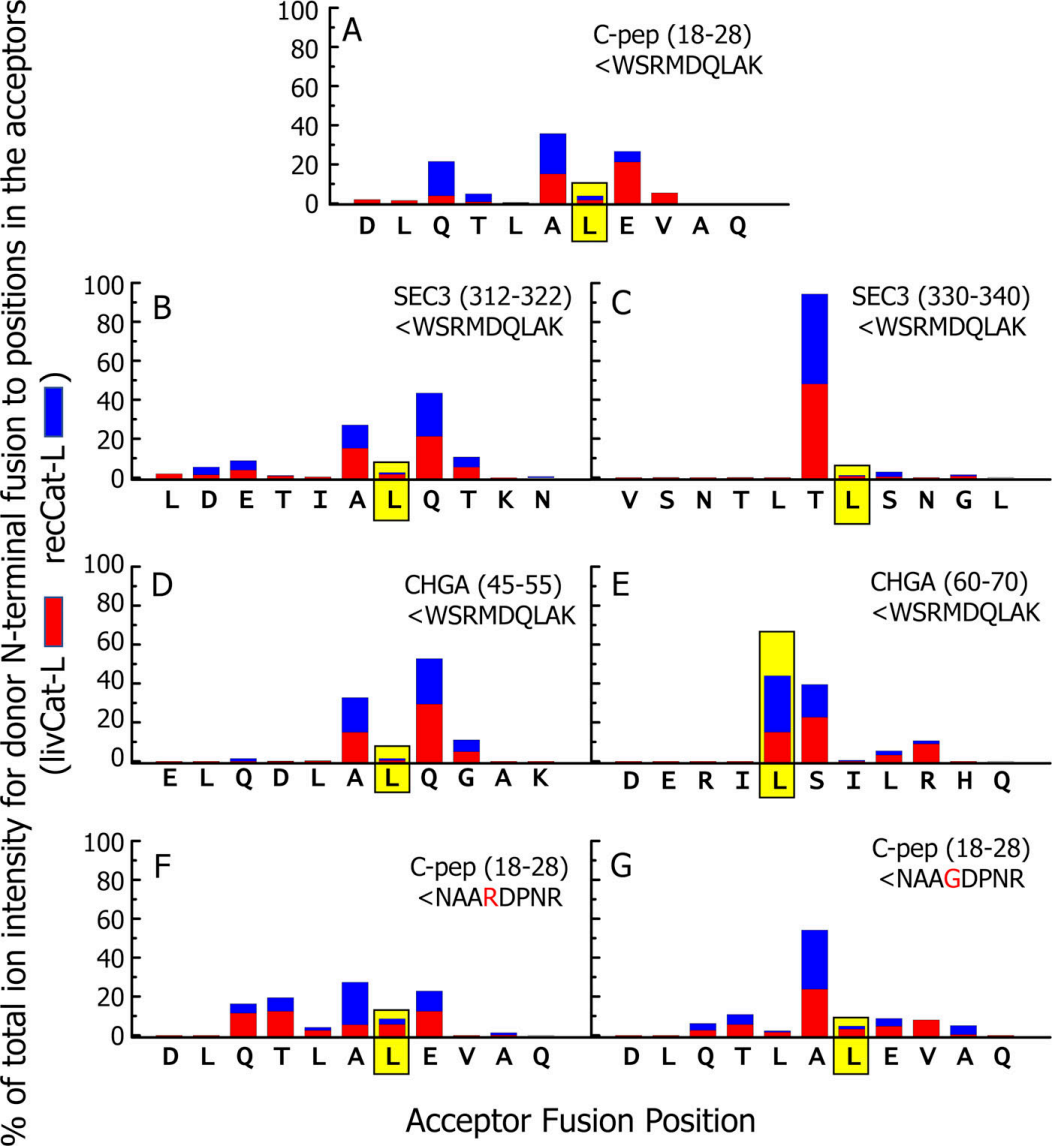
**Extensive analysis of the transpeptidation fusions within the digests are consistent with the cleavage properties of Cat-L.** Data S4 lists all of the chimeric peptides obtained with either Cat-L preparation in which the N terminus of the input donor peptide was found fused to any position in the acceptor peptide. This figure summarizes the data in Data S4. **(A–G) **Each figure shows data for digestions using one combination of acceptor and donor and either livCat-L (red) or recCat-L (blue) in the digestions. The sequence of the amino acids in the acceptor peptide that could be sites for transpeptidation are shown along the x axis. The sums of the ion intensities of chimeric peptides identified at each fusion site regardless of truncations at either end were calculated separately for each Cat-L and are expressed as the percentage of the total ion intensities at all sites. The graph shows the percentages obtained with either Cat-L divided by 2, stacked and plotted versus the fusion amino acid on the x axis. The donor peptide is shown in the upper right position of each figure. The data for the fusion position that generated the functional epitopes are framed in yellow.

Publications going back >80 yr show that transpeptidation is an inevitable side reaction in any proteolytic digestion involving serine, threonine, or cysteine proteases due to the ability of the N terminus of nearby peptides to replace water in resolving the covalent peptide–protease intermediate (reviewed in Berkers et al., 2009). Therefore, to examine how extensive the transpeptidation reaction was in our digestions, we looked at the chimeric peptides present other than just the ones that contained the predicted functional epitope. We examined all of the peptides that involved the N terminus of the input donor peptide fused to any site within the input acceptor peptide (Data S4). Among the 14 digestions, of the possible 70 total input acceptor fusion sites, 68 were found fused to the N terminus of the input donor peptide with at least one of the Cat-Ls, and 46 occurred with both proteases. We used the total ion intensities of all peptides with a particular fusion site, regardless of length, as an approximation of the efficiency of transpeptidation at that site. These values were plotted versus the position in the acceptor peptide for both preparations of Cat-L (Fig. 8, A–G).

These results revealed several features of the Cat-L–mediated transpeptidation mechanism. First, the patterns with the livCat-L and recCat-L were very similar, again indicating that the active protease in the liver preparation was in fact Cat-L. Second, the most efficient fusion sites were clustered in the middle of the acceptor peptide, with very inefficient fusions to the first two or last two potential sites in the acceptor, suggesting that the cleavage reaction is most efficient when approximately six to seven amino acids fill the Cat-L binding site. This agrees with preferred endopeptidase property of Cat-L and its peptide binding cleft (Sosnowski and Turk, 2016). Third, defining the protease recognition sequence as p4-p3-p2-p1-p1′-p2′-p3′-p4′, with the cleavage site after the p1 amino acid, the most frequent sites of transpeptidation were positions in the acceptor peptide with an L or I at p2 and any of several amino acids at p1. This correlates well with the published literature for Cat-L (e.g., Kirschke, 1981) that shows, despite some promiscuity in the cleavage sites for the protease, especially at acidic pH, there is preference for an aliphatic (L, I, or V) or aromatic (F or Y) amino acid at p2 (Rawlings et al., 2018). This promiscuity was essential for generating the functional chimeric peptides in our experiments, since, except for the ChgA acceptor cleaved at ERIL, none of the functional fusions we detected involved a favored Cat-L cleavage site.

Our analyses shown in Data S4 revealed hundreds of other chimeric peptides in our digestions besides those described in Fig. 8, involving fragments of the input acceptor and donor peptides fused together in either direction. With a few exceptions, these had low ion intensities and lower confidence scores. The results with the two versions of Cat-L were overlapping but not identical. However, it is worth noting that, as with the chimeric peptides described in Fig. 8, the fusions tended to occur at sites favored by Cat-L, i.e., an aliphatic amino acid at p2 and many other amino acids at the p1 cleavage site. For example, among the >1,000 additional peptides found in the digestions with a mixture of various peptides and WSRMDQLAK, >40% involved a fusion with L, I, or V at the p2 position. Taken together, these data highlight how robust the Cat-L transpeptidation reactions were under these lysosomal conditions.

## Discussion

Originally, deletion of self-reactive T cells was shown to occur in the thymus during development (Kappler et al., 1987; Kisielow et al., 1988). At the time, not all self-peptides were thought to be expressed in the thymus, so how could T cells specific for such peptides be deleted in the thymus? The discovery of ectopic expression of peripheral-organ-specific proteins in thymic epithelial cells expressing the autoimmune regulator (*AIRE*) gene provided a mechanism for negative selection of such cells (Anderson and Su, 2011). However, parallel experiments showed that thymic negative selection was insufficient to establish self-tolerance completely, and the peripheral action of self-reactive regulatory T (T reg) cells was needed to control the thymic escapees (Chatila, 2005; Curotto de Lafaille and Lafaille, 2002). Thus, the nature of the CD4 T cell epitopes driving T1D and other autoimmune diseases remained a puzzle, since these T cells appear to have escaped both control mechanisms. The importance of posttranslationally created epitopes in the periphery for CD4 T cells in rheumatoid arthritis (Wegner et al., 2010) and celiac disease (Sollid and Jabri, 2011) gave hints to mechanisms for how T cells escape both thymic deletion and peripheral T reg cells to go on to cause autoimmunity. If the epitope were confined to the target organ, thymic deletion would not be possible, and the inflammation initiated in the organ by T cells recognizing highly immunogenic neo-antigens could overwhelm the T reg cell mechanisms.

Creation of chimeric neo-epitopes for CD4 T cells by transpeptidation in the target tissues would further support these ideas. The milieu for transpeptidation is particularly favorable in T1D and other autoimmune diseases of neuroendocrine tissues, where tissue-specific proteins are packaged at high concentrations in secretory granules. Intracellular levels of secretory granules are highly regulated in these tissues, and excess granules are turned over by crinophagy, i.e., catabolic fusion of granules with lysosomes (Weckman et al., 2014). Crinophagy sets up the perfect conditions for transpeptidation, namely, high concentrations of the granule proteins exposed to a variety of cysteine and serine proteases in organelles with low water content and an acidic pH. Moreover, several studies have shown that the rate of crinophagy is enhanced by conditions that induce cell stress, typically seen in β cells during the onset of diabetes (Sandberg and Borg, 2006). These inflammatory conditions could also help T cells escape the action of T reg cells. On the other hand, it is unlikely that the conditions to produce these chimeric peptides exist in the thymus. Despite the fact that the genes for proteins found in neuroendocrine cells can be found ectopically expressed in some thymic *AIRE*^+^ cells, so far this detection has been confined to mRNA, not protein. Given the limited number of *AIRE*^+^ cells among thymic medullary cells, characterization of their MHCII-bound peptide repertoire is not possible with present methods. However, the lack of neuroendocrine-like secretory granules in these cells would make it unlikely that the concentrations of the required components for creating the chimeric peptides would ever be achieved in their lysosomal/endosomal compartment. Furthermore, in those cases in which the donor and acceptor required to form the functional chimeric epitope come from different proteins, e.g., C-peptide and WE14, it is highly unlikely that a given *AIRE*^+^ cell would express both proteins at the same time.

Our experiments reported here show that transpeptidation mediated by Cat-L, and likely by other lysosomal proteases as well, is very efficient in generating self-protein–derived chimeric epitopes for potential MHCII presentation to CD4 T cells, including diabetogenic autoreactive T cells. These results complement the recent work on the role of transpeptidation by proteasomal proteases in generating epitopes for MHCI presentation to CD8 T cells. Together, they point out that using the standard transcriptomes for identifying self-peptides isolated from MHCI and MHCII would miss these important chimeric peptides. The recent computational methods for screening MHCI-bound peptidomes for chimeric peptides have shown that a substantial proportion of peptides are in fact chimeric peptides formed by proteasomal transpeptidation within a protein or between different proteins (Faridi et al., 2018; Liepe et al., 2016). While the great variation in the lengths of peptides found bound to MHCII versus MHCI makes this type of analysis much more difficult, it is likely that similar approaches will eventually be developed for MHCII-bound peptides. Our results with Cat-L predict that chimeric peptides will be common among these peptidomes, pointing to the lysosome as a potent source of these peptides.

Our results show that in vitro, Cat-L is sufficient for the generation of a number of chimeric diabetogenic epitopes from suitable donors and acceptors. They do not establish whether Cat-L is necessary for generating these chimeric epitopes; i.e., can other proteases create them as well? There are several studies relevant to this question. They show that genomic ablation of Cat-L protects NOD mice from developing T1D (Hsing et al., 2010; Jung et al., 2015; Maehr et al., 2005). However, the authors conclude that other mechanisms are likely at play, e.g., interference with thymus selection and/or diversion of CD4 T cells into the T reg cell pathway, rather than the loss of processing the diabetic epitopes. In fact, one of these studies (Maehr et al., 2005) demonstrates that a functional, but unknown, ligand for the BCD-2.5 T cell is still present in the pancreatic lymph nodes of Cat-L^−/−^ NOD mice. Cat-L and other cathepsins play a general role in antigen and invariant chain processing in thymic epithelial cells and other APCs. Other confounding factors are the redundant activities among the many lysosomal cysteine/serine proteases and the multiple different chimeric epitopes that stimulate a given CD4 T cell that we show here and previously (Wang et al., 2018). Therefore, isolating the role of a particular cathepsin in the creation of particular chimeric epitopes in situ in mice or β cell lines with gene knockouts and inhibitors is not feasible at this time.

We have proposed that transpeptidation may also play a role in the CD4 T cell response to the insulin B chain peptide, B:9-23 peptide, in T1D in mice and humans (Jin et al., 2015; Wang et al., 2018, 2019). We have shown that the poor presentation and recognition of this peptide by mouse IA^g7^ and human HLA-DQ8 can be vastly improved by changing amino acids at its C terminus at B:21 (p8) and/or B:22 (p9; Crawford et al., 2011; Marrack and Kappler, 2012; Stadinski et al., 2010b; Wang et al., 2018; Yang et al., 2014). Furthermore, these mutations can also be accomplished by synthetic fusion of fragments from proinsulin C-peptide to the B chain peptide truncated to B:20 or B:21 (Wang et al., 2019). Therefore, we have proposed that these fusions could also be achieved by internal transpeptidation within proinsulin. However, to date we have not identified a lysosomal protease that in our hands cleaves proinsulin at the appropriate site in the B chain to allow us to test this idea, but we have not yet made an exhaustive search.

If transpeptidation modifications of proinsulin, ChgA, and IAPP within the β cell generate the chimeric epitopes for diabetogenic CD4 T cells, how do these peptides find their way to APCs to become the MHCII-bound ligands for these T cells? The answer may lie in recent experiments by others showing that antigen-containing exosomes derived from β cell crinophagic bodies are shed as exosomes from the β cell and can be taken up by APCs for presentation to T cells (Wan et al., 2018). These exosomes would be an excellent place to search for the chimeric peptides. In summary, our data presented here open the door for delving more deeply into the search for chimeric MHCII autoantigen epitopes derived from secretory granule proteins during catabolic crinophagy, not only in T1D, but also in autoimmune diseases involving other neuroendocrine tissues.

## Materials and methods

### Peptide synthesis

Synthetic soluble peptides (>95% pure) were obtained from CHI Scientific, Schafer-N, or Genscript.

### Soluble TCR and MHCII proteins

Soluble BDC-2.5 TCR and IA^g7^-peptide complexes were produced as previously described in baculovirus-infected High5 insect cells (Invitrogen) using a two-promoter baculovirus transfer vector (Kappler et al., 1994; Kozono et al., 1994). Briefly, for the BDC-2.5 TCR, the genes encoding the extracellular portions of the TCR α and β chains were cloned after the promoters (P10 for α and polyhedron for β) in the transfer vector. For use in flow cytometry, the genes were further modified by adding acid-base leucine zippers to the ends of the C-regions (basic to Cα and acidic to Cβ), and a peptide tag for biotinylation by the biotin-protein ligase (BirA) enzyme was added to the C terminus of the acidic zipper (Crawford et al., 1998). For expression, the transfer vectors were cotransfected into SF9 cells with linearized baculovirus DNA. Successful recombination of the transfer vector into the baculovirus DNA in the SF9 cells resulted in circular infectious DNA and expression of the soluble TCR. The virus was cloned and expanded in SF9 cells to prepare a viral stock for infection of High5 insect cells for production of the soluble TCRs, which were purified from the culture supernatants by affinity chromatography (Crawford et al., 1998). The TCR bearing the peptide tag for the BirA enzyme was biotinylated by the enzyme, complexed with streptavidin-Brilliant Violet 421, and separated from the uncomplexed TCR by Superdex S200 size exclusion chromatography (Crawford et al., 1998).

Genes for the soluble versions of IA^g7^ bound to RLGL–WE14, SRLGLWVRME, or a negative control HEL peptide and with acid-base zippers and a BirA biotinylation tag were cloned in the same two-promoter baculovirus transfer vector and similarly recombined into linearized baculovirus DNA. The resulting virus was cloned, expanded, and used to express the soluble IA^g7^ complexes, which were biotinylated with BirA (Crawford et al., 1998).

### SPR

Interactions of the unbiotinylated soluble BDC-2.5 TCR with the biotinylated IA^g7^-peptide complexes were evaluated using a BIAcore 2000 instrument (GE Healthcare). Approximately 2,000 RU of each of the three biotinylated IA^g7^ complexes were captured in separate flow cells of a streptavidin BIAsensor chip. Various concentrations of soluble BDC-2.5 TCR were injected through the flow cells, and the binding to and dissociation from the IA^g7^ complexes were recorded as the RU signal, which was used to calculate the binding kinetics using BIAevaluation 4.1 software (see Fig. 1 B legend for more details).

### Baculovirus IA^g7^-peptide library with four randomized amino acids linked to WSRMDQ

We prepared a baculovirus library encoding peptides linked to IA^g7^ by a variation of our published methods (Crawford et al., 2004, 2006; Stadinski et al., 2010a; Yin et al., 2012). Briefly, the IA^g7^ genes cloned into the two-promoter baculovirus described above were used to construct full-length circular baculovirus DNA encoding an altered version of the IA^g7^ β chain. In the construct, the C-terminal BirA biotinylation tag was replaced with DNA encoding the transmembrane and cytoplasmic tail of the baculovirus gp64 protein. In addition, an SceI homing enzyme site was placed before baculovirus polyhedrin promoter. The β chain signal peptide, peptide, and part of the linker to the β chain N terminus was replaced with the gene for RFP followed by a termination codon and a site for the CeuI homing enzyme. This construct was introduced into baculovirus DNA as above. When infected with the resulting virus, SF9 cells expressed RFP but no detectable IA^g7^. Digestion of the DNA with the homing enzymes created a noninfectious linear baculovirus DNA with different four-base overhangs at the 3′ and 5′ ends of the restricted DNA.

Meanwhile, a DNA fragment was produced encoding a BstXI restriction site followed by the baculovirus polyhedrin promoter, a translation initiation codon, ATG, the β chain signal peptide, four randomized amino acids, the N-terminal fragment of WE14 (WSRMDQ), and sequence for the missing linker amino acids, followed by a second BstXI site. The six bases interrupting the BstXI palindrome at the two ends of the fragment were different such that, when cut with BstXI, unique overhangs were generated compatible with the homing enzyme SceI and CeuI sites in the digested baculovirus DNA. The randomized amino acids were encoded by NN(G/C), where N is an equal proportion of all four bases and (G/C) indicates an equal proportion of guanosine or cytidine. The synthesized fragment was sequenced to confirm that the codons for the randomized amino acids were as expected.

The baculovirus DNA restricted with the SceI/CeuI homing enzymes was ligated with an excess of the BstXI restricted fragment, thereby restoring the polyhedrin promoter, while fusing sequence encoding the signal peptide, randomized amino acids, and WSMRMDQ, as well as additional linker amino acids which were frame to the IA^g7^ β chain gene. Since the ligation also circularized the baculovirus DNA to an infectious form, transfection with this ligated DNA into SF9 cells led directly to virus encoding surface IA^g7^, with each virus expressing an IA^g7^ covalently linked to a different member of the peptide library. For the experiments described in Fig. 1 C, we estimated that this procedure generated a library of ∼10^7^ independent viruses.

### Flow cytometry

Flow cytometry was performed with a MoFlo II instrument (Dako/Cytomation) for sorting and a CyAn flow cytometer (Dako/Cytomation) for analysis.

### T cell hybridomas and antigen presentation assays using soluble peptides

For APCs, we used the IA^g7^-expressing M12.C3 B cell lymphoma (M12.C3.g7; Carrasco-Marin et al., 1996). Four T cell hybridomas were used in various stimulation assays. BDC-2.5 and BDC-10.1 were provided by Dr. Kathryn Haskins (University of Colorado Medical School, Aurora, CO). G7W120 was produced in this laboratory (Jin et al., 2015). The insulin B:9-23 reactive AS91 T cell hybridoma (Levisetti et al., 2007) was provided by Dr. Emil Unanue (Washington University, St. Louis, MO). T cell hybridomas (10^5^ cells) were mixed with either 10^5^ paraformaldehyde-fixed APCs or 10^5^ unfixed APCs and cultured overnight with various concentrations of synthetic peptide or volumes of a pH-adjusted cathepsin digestion mixture in a final volume of 250 µl. Secreted IL-2 was assayed with a functional assay where the growth and survival of an HT-2 IL-2–dependent cell line was quantified using the dye MTT (White et al., 2000) and reported as units per milliliter based on comparison to an IL-2 standard.

### Cathepsin digestion reactions

Cathepsin digestions of peptides and proteins were performed by variations of methods previously reported for in vitro cathepsin-mediated proteolysis (Kim et al., 2014; Poluektov et al., 2013) and adapted for our studies. Briefly, digestions contained 20 µl of 0.1 M citric acid/Na phosphate buffer (pH 5.0) containing 6 mM L-cysteine and 4 mM EDTA, as well as the protein or peptide substrates and a human cathepsin protease (livCat-L, C6854; livCat-B, 219362; or recCat-S, 219343; Millipore-Sigma) or recCat-L prepared in *E. coli* (1135-100; BioVision). Digestions were for 60 min at 37°C followed by neutralization with 20 µl of 0.02 M NaH_2_PO_4_, pH 9.15. 20 µl of the reaction was removed for testing in the T cell stimulation assay. Iodoacetamide was added to the remaining 20 µl of the digest at 12 mM for 30 min on ice in the dark to inactivate the cathepsin. These samples were stored at −80°C until MS/MS analysis.

The amounts of substrates and cathepsins in the digestions are listed in the legends of the figures describing the results for that digestion. In some digestions, various concentrations of the specific Cat-L inhibitor, RKLLW_NH2_ (SCP0110; Sigma-Aldrich), was added. In these cases, Cat-L was preincubated with various concentrations of Cat-L inhibitor for 30 min on ice, before adding to the reaction. All of the digestions that appear in the manuscript article were done at either a 1:3 or 1:10 acceptor:donor ratio in order to favor the transpeptidation reaction. Additionally, each enzyme used was titrated to determine the optimal concentration for each reaction. In the experiments presented here, ultimately 7 ng of livCatL (based on its actual concentration in the preparation determined above) or 12.5 ng of recCatL were used for all of the digestions.

### Proteomic analysis: liquid chromatography (LC)–MS/MS

Equal molar amounts of all samples were desalted and prepared for LC-MS/MS analysis as previously described (Dempsey et al., 2019). Briefly, samples were desalted using stage tips packed with styrene divinyl benzene (Empore) extraction discs. Desalted samples were brought up to ∼1 pmol/µl target peptide in 0.1% formic acid. LivCatL samples were then analyzed on a LTQ Orbitrap Velos Pro MS (Thermo Fisher Scientific) coupled with an Eksigent nanoLC-2D system. Approximately 5 pmol of each sample target was injected onto a trapping column (ZORBAX 300SB-C18, 5 µm) and washed with 2% acetonitrile and 0.1% formic acid for 5 min. The trapping column was then switched online with the nanopump at a flow rate of 350 nl/min. Peptides were separated on an in-house-made 100 µm × 180 mm fused silica capillary packed with 2.7 µm Phenomenex Cortecs C18 resin over a 20-min gradient from 2 to 32% acetonitrile. Additional gradient consideration and MS acquisition parameters were described previously (Hill et al., 2015), with the exception that samples were acquired with a higher collision energy dissociation at an activation energy of 35. RecCatL samples were analyzed on a Q Exactive HF mass spectrometer (Thermo Fisher Scientific) coupled to an EASY-nanoLC 1000 system. Approximately 5 pmol of each sample target was injected onto an analytical column with the same specifications as above at a flow rate of 400 nl/min over a 30-min gradient from 2 to 32% acetonitrile. Additional MS acquisition parameters were described previously (Carlson et al., 2019).

Raw files were directly loaded into Proteome Discoverer 2.2 and searched against an in-house-generated database consisting of all theoretically possible fusion peptides in addition to common contaminants using the Mascot search engine. Mass tolerances were set to ±10 ppm for MS parent ions and ±25 ppm for MS/MS fragment ions. No enzyme specificity was set, to allow for any possible cleavage variant. Met oxidation and peptide N-terminal pyroglutamic acid formation were set as possible variable modifications. Peptides meeting a 0.01 false discovery rate threshold were considered for this analysis. The data for identified peptides and the fragmentation spectra were exported into an Excel spreadsheet for further analysis as described in Results, and graphical representations of the data were generated using Grapher 12.7 (Golden Software).

### Online supplemental material

Fig. S1 shows the four amino acid peptides that, when fused to WSRMDQ fragment of WE14, have weak or no activity in stimulating WE14-specific T cells (in support of Fig. 2). Fig. S2 depicts how the commercial human livCat-L is contaminated with other proteins, including several other cathepsins in addition to Cat-L (in support of Figs. 3, 4, 5, 6, 7, and 8). Fig. S3 shows how the MS/MS fragmentation patterns for the seven full-length functional chimeric peptides generated with livCat-L match those for synthetic versions of the same peptides (in support of Figs. 6 and 7). Data S1 lists the library peptides with a randomized four–amino acid N-terminal extension to WE14 selected by the BCD-2.5 TCR. Data S2 lists the fragments of human proinsulin and mouse C-peptide obtained by digestion with Cat-L. Data S3 lists all of the possible chimeric peptides that could be formed between the two input peptides in the Cat-L digestions in described Figs. 4 and 7. Data S4 lists all of the chimeric peptides identified in the Cat-L digestions described in Figs. 4 and 7. Data S5 lists all of the potential y and b fragments that could be generated during MS/MS analyses of the full-length functional chimeric peptides identified in Figs. 4 and 7.

## Supplementary Material

Data S1 lists the library peptides with a randomized four–amino acid N-terminal extension to WE14 selected by the BCD-2.5 TCR.

Data S2 lists the fragments of human proinsulin and mouse C-peptide obtained by digestion with Cat-L.

Data S3 lists all of the possible chimeric peptides that could be formed between the two input peptides in the Cat-L digestions in Figs. 4 and 7.

Data S4 lists all of the chimeric peptides identified in the Cat-L digestions described in Figs. 4 and 7.

Data S5 lists all of the potential y and b fragments that could be generated during MS/MS analyses of the full-length functional chimeric peptides identified in Figs. 4 and 7.
